# Bad Colds

**Published:** 1871-06

**Authors:** 


### HALL'S

### JOURNAL OF HEALTH.

VOL. XVIII. — JUNE, 1871. — NO. VI.

### BAD COLDS.

A COMMON cold always arises from some part of the skin becoming cooler than natural, and remaining so for a greater or less time; the longer the time, the more severe the cold; the greater the surface thus cooled, the more severe the cold, other things being equal. The natural heat of the skin is about one hundred degrees; common water, of a summer's day, is about fifty degrees; hence if a piece of muslin or other material is dipped in water and laid on the skin and kept there for a while in that moist condition, a cold will follow.

If a person, exposed to a shower of rain, is "wet to the skin," and sits still until the clothing has dried, a cold follows; but if the same person keeps in active exercise until the clothing is dried, no cold follows, because the exercise keeps the skin warm, keeps the wet garment warm, and soon dries it. But a fever always follows a cold; that is, if the skin is kept colder than is natural for a while, it inevitably becomes warmer than natural; this is the reaction. If the pendulum of a clock is drawn up one side and then let go, it will go very nearly as far on the other side, but not just as far: at each swing, whether on one side or the other, this pendulum comes not quite to the point it left; so losing something at each swing, it finally comes to a stop, unless there is some outside contrivance to keep it going.

So in an ordinary cold: there is chill first, then fever, each in time becoming less and less, until an equilibrium is restored; until there is no cold above what is natural, and no heat above what is natural. Hence a cold will cure itself in ordinary cases if it is let alone; for as it is "natural" for a pendulum to come to a stand-still when swung once from the finger, that is, to come to a state of rest or equilibrium, so it is "natural" for the body to come to its state of rest or equilibrium of heat and cold.

But a cold may be cured; that is, this alternation of chill and fever, of too much cold and too much heat, may be modified, may be made to become less and less until it disappears altogether, by applying cold to the fevered skin, and warmth to the chilled skin; a better plan, however, is when the skin is warm to keep it so; then a chill cannot follow; and this is done by putting the skin in a perspiration and keeping it in that condition through the time that the chill would have lasted, say an hour or two, or more, according to the severity of the case. So, after all, the best, the most philosophical cure in the world for a cold, is the remedy of our great-grandmothers, — "a sweat." All of us know what an ugly chillness runs over us, especially up and down the back, when we have a cold; how we hug up to the fire and turn round before it, warm on one side, cold on the other; and then before we know it, we are hot and feverish, and nature calls for cold water to cool off the fever; and we drink and drink and drink until all thirst is gone, but the fever remains, and the best ice-water has a metallic taste.

There is another method of dealing with a cold, which, if adopted, would prevent in New York city alone, in one year, myriads of days of illness, of unfitness for business and enjoyment. In the healthy state of the human body, there are millions of little streams of water flowing from within the body to the outside, amounting in all, in a day, to a pint or two of water. This water is full of the impurities, the wastes of the system; that is, Nature is all the time washing out the house we live in; the instant the skin is chilled, the outlets of these little streams, called

"PORES OF THE SKIN,"

are closed, are contracted (cold causes all bodies to contract), and the impurities are kept within the body, — are mixed up with the blood, making it impure, thick, and unnatural; being thick, it does not flow well through the body, in fact "dams up," congests in various parts, filling them fuller of blood than they ought to be, causing a general feeling of discomfort all over the body. The lungs being delicate and distensible, and having the least power of resistance, are the readiest points for the accumulations, congestions, or damming-up process, already alluded to, of bad, impure blood; and being "oppressed," — and who does not feel oppressed under the influence of a "bad cold?" — Nature makes an effort to relieve herself, and does it thus: the blood in the lungs is turned to "phlegm," a thick, yellowish material; this is made very gradually, and by slow degrees is pushed up to the top of the lungs, from all directions, to the bottom of the windpipe, answering very nearly to the little hollow at the bottom of the neck, or top of the breast bone, the very spot where a "crumb" or a drop of water goes, when it "goes the wrong way," and we all know what an urgent coughing such a thing induces; at this point then, the mattery substance accumulates, and causes a tickling similar to that induced by a crumb of bread or a drop of water going the wrong way; this tickling causes cough, which is the forcible expulsion of air from the lungs, carrying out everything before it into the mouth, from which it is expectorated. But suppose there was no cough, this matter would accumulate very steadily in the lungs until they would become so full, that the air could not get in at all, and we would inevitably suffocate. It is demonstrable then that

"COUGH CURES A COLD,"

by relieving the lungs of what does not belong there; and yet, nine persons out of ten are constantly taking something for the cough; that is, are taking something which will prevent coughing, and that which prevents it soonest and most effectually is said to

"WORK LIKE A CHARM;"

thus actually keeping in the body that ugly yellow matter which the cough brings up, and which, when out, we look upon with disgust, and would fairly loathe to touch with the fingers. What is it that causes

"THE HECTIC HUE OF DEATH,"

the red spot on the cheek of the consumptive, which tells too surely that his race is run? It is the yellow matter which, failing to be brought from the lungs fast enough by the cough, is re-absorbed, carried back into the blood, poisons it, and he dies.

Hence, the more a man coughs up, who has a cold, the sooner he will get well; and really, instead of taking something to cure his cough, it would be better to do something which would make him cough more. Isn't man the biggest fool in the universe! The world is six thousand years old, and yet it has not been announced before in print, to the best of our knowledge and belief, that in curing a common cold, it is better to increase the cough than to repress it.

STARVE A COLD.

It has been shown that a cold makes phlegm; that this phlegm is made out of an excess of blood in the lungs; and as the food we eat is made into blood, it follows that the less blood is made, the less phlegm there is to be coughed up; therefore the less a man eats when he has a cold, the less blood is made, the less phlegm is made, the sooner the cough ceases, and the sooner he gets well. And yet, when we take cold, we go on eating as before; when, in reality, every mouthful swallowed protracts the cure of the cold by that much; by that much makes more coughing necessary.

A cold is essentially too much blood in the body — blood loaded with impurities: the rational mode of cure is to diminish the amount of blood; relieve the system of its accumulations, either by coughing up the yellow matter from the lungs, by promoting perspiration, or by causing the bowels to act more freely, as by a large dose of castor oil or salts; or by stopping the manufacture of more blood by ceasing to take more food into the system, out of which food the blood is made; not by ceasing to eat altogether longer than twenty-four hours, but after fasting, eating nothing that long, take only gruel. Oatmeal gruel or porridge is best, at five hours' interval, from sunrise to sunset.

As medical observation seems to show that the most fatal consumptive colds are taken in summer, this is a "seasonable" topic for June.

